# Adventitious Viruses and Smallpox Vaccine

**DOI:** 10.3201/eid1111.051031

**Published:** 2005-11

**Authors:** Claude Chastel

**Affiliations:** * Virus Laboratory, Brest, France

**Keywords:** smallpox vaccine, adventitious viruses, risk, letter

**To the Editor:** Recently, Murphy and Osburn (*1*) strongly argued for testing old smallpox vaccine stocks made in animal skin for adventitious infectious agents such as viruses, mycoplasmas, and eventually, prions. Their argument appears clearly justified after unexpected cases of myopericarditis occurred during recent campaigns of smallpox vaccinations in the United States (*2*).

To the long list of bovine viruses cited in this paper, it seems necessary to add another, the pseudocowpox virus, a widespread parapoxvirus that may infect humans. During the 1960s, this virus was identified in vaccine lymph from a heifer at the Institut Pasteur, Paris (*3*).

In humans, this virus is responsible for limited skin lesions, more frequently in immunocompromised patients. Mainly farmers and butchers are affected. Pseudocowpox virus is easily differentiated from orthopoxviruses such as vaccinia virus by the virus's peculiar form on transmission electron microscopy scan, but polymerase chain reaction is probably the best detection method (*4*). In fact, many other more hazardous viruses may be found in the oldest stocks of smallpox vaccine and deserve more attention than previously considered.
